# Diverse TRPV1 responses to cannabinoids

**DOI:** 10.1080/19336950.2019.1619436

**Published:** 2019-06-04

**Authors:** J. Starkus, C. Jansen, L. M. N. Shimoda, A. J. Stokes, A. L. Small-Howard, H. Turner

**Affiliations:** a Laboratory of Immunology and Signal Transduction, Chaminade University, Honolulu, HI, USA; b Laboratory of Experimental Medicine, John A. Burns School of Medicine, Honolulu, HI, USA; c GBSciences, Inc., Las Vegas, NV, USA

**Keywords:** TRP channels, cannabinoids, pain, calcium, oxidation

## Abstract

Cannabinoid compounds are potential analgesics. Users of medicinal *Cannabis* report efficacy for pain control, clinical studies show that cannabis can be effective and opioid sparing in chronic pain, and some constituent cannabinoids have been shown to target nociceptive ion channels. Here, we explore and compare a suite of cannabinoids for their impact upon the physiology of TRPV1. The cannabinoids tested evoke differential responses in terms of kinetics of activation and inactivation. Cannabinoid activation of TRPV1 displays significant dependence on internal and external calcium levels. Cannabinoid activation of TRPV1 does not appear to induce the highly permeant, pore-dilated channel state seen with Capsaicin, even at high current amplitudes. Finally, we analyzed cannabinoid responses at nociceptive channels other than TRPV1 (TRPV2, TRPM8, and TRPA1), and report that cannabinoids differentially activate these channels. On the basis of response activation and kinetics, state-selectivity and receptor selectivity, it may be possible to rationally design approaches to pain using single or multiple cannabinoids.

## Introduction

Chronic pain debilitates millions of persons worldwide. Median prevalence estimates suggest chronic pain patients are ~15% of the US population and at least 116M US persons have a pain condition [1]. Economic estimates of the healthcare burden associated with pain vary, but the Institute of Medicine estimates the national economic cost of chronic pain to be between $560-635B annually [2]. Pain presents on a clinical spectrum ranging from mild acute pain for which over-the-counter (OTC) medications are sufficient, to acute, recurring episodes and chronic debilitating pain that requires constant medication. In the latter two categories, major shortfalls in pain medication strategies exist: (1) the tendency for relatively benign, non-addictive treatment modalities to become ineffective over time, due to receptor desensitization and cellular adaptation, and (2) the addictive nature of highly efficacious pain medications such as opioids [3–5].

There is a need for pain medications that are mechanistically likely to be able to address chronic pain as well as acute presentations, by acting analogously to current methods for desensitization of nociceptive neurons and pathways. Analgesics that are likely to be non-addictive and have a low side-effect profile are being actively sought. Traditional medicine from many cultures suggests that plants are possible sources of complex chemical mixtures that can be used to treat diverse human conditions, including pain [6–13]. The secondary metabolome of the *Cannabis* plant has been suggested as sources of new analgesics [14–17]. Pain is a primary use of medicinal *Cannabis* and substitution for prescription opioids is common [18,19], and demonstrably opioid sparing [20–22]. Unregulated “medical marijuana” use for pain covers multiple demographics and disorders, even reaching historically underserved groups such as seniors facing undertreatment of pain at end-of-life [23]. However, safety, efficacy, and consistency of plant-derived medicines such as medical *Cannabis* do not yet approach traditional pharmaceutical standards for widespread therapeutic deployment [16,24–28]. Thus, there is a need for well-defined compositions of secondary metabolites from *Cannabis* that selectively relieve pain. Excluding the psychoactive tetrahydrocannabinol, which is not a desirable approach for therapy [29], major cannabinoids (cannabidiol, cannabinol), minor cannabinoids (e.g., cannabichromene, cannabigerol, cannabividarin), and various terpenes (e.g., myrcene) are all potential sources of pain management if their efficacy at nociceptive targets can be proven [30].

Channels of the Transient Receptor Superfamily (TRP), such as TRPV1, TRPM8, and TRPA1, are non-selective cation channels that conduct calcium and sodium into a range of cell types in mammals. They are present on sensory neurons, and were initially identified as having a role in nocioception because of their responsiveness at the molecular level to plant secondary metabolites that are nocimimetic (e.g., Capsaicin) and to compounds that are otherwise pungent and mimic burning or cooling sensations (e.g., allicin, cinnamaldehyde, menthol) [31–36]. Several of these channels are targets for cannabinoids including THC, CBD and CBN and some minor *Cannabis* compounds [37–44]. However, the impact of all these compounds at TRPV1 has not been systematically evaluated using electrophysiological techniques, and their commonalities or differences to standard Capsaicin treatment have not been fully established. Moreover, the potential, or otherwise, of cannabinoids to selectively target, or co-target channels that mark specific neurons in a sensory bundle has not been evaluated.

Both antagonism and agonism of the TRP channel are critical pharmacological approaches for pain management [45–48]. For example, TRPV1 antagonism has utility in acute analgesia but chronic pain management requires longer-term strategies such as receptor and neuronal desensitization using TRPV1 agonists. In practice, this therapy applies high levels of Capsaicin topically, and repeatedly over time to the affected area, a painful process in its own right [49–52]. If cannabinoids represent an approach for pain then there is a need to evaluate their activation, inactivation and desensitization behavior compared to that of Capsaicin.

In this study, we explore and compare a suite of cannabinoids for their impact upon the physiology of TRPV1. The cannabinoids tested evoke differential responses in terms of kinetics of activation and inactivation. Cannabinoid activation of TRPV1 displays significant dependence on internal and external calcium levels. Cannabinoid activation of TRPV1 does not appear to induce the highly permeant, pore-dilated channel state seen with Capsaicin, even at high current amplitudes. Finally, we analyzed cannabinoid responses at nociceptive channels other than TRPV1 (TRPV2, TRPM8, and TRPA1), and report that cannabinoids differentially activate these channels. On the basis of response activation and kinetics, state-selectivity and receptor selectivity, it may be possible to rationally design approaches to pain using single or multiple cannabinoids.

## Materials and methods

### Cell culture

HEK TRexTRPV1 [53] were cultured in DMEM, 10% Fetal Bovine Serum, 2mM L-glutamine, 10 μg/ml Blasticidin (Calbiochem, San Diego CA), 400 μg/ml Zeocin (InvivoGen, San Diego CA), where indicated transgene expression was induced using 1 μg/ml Tetracycline for 16–24 h. Unless otherwise indicated, basal expression of TRPV1 without induction was sufficient for these studies, and comparisons were made to untransfected HEK where needed. HEKTRex293 over-expressing human TRPV2, human TRPA1, and human TRPM8 were obtained from SB Drug Discovery (Glasgow, Scotland) and cultured as described above.

### Chemicals, reagents, and stimulations

General chemicals were from VWR (West Chester, PA) and Sigma Aldrich (St. Louis, MO). PMA and Ionomycin were from Calbiochem (Gibbstown, NJ). IgE anti-DNP is from Sigma and KLH-DNP was from Calbiochem. Capsaicin and Capsazepine were from Sigma Aldrich. Cannabidivarin (CBDV), Cannabichromene (CBC), Cannabidiol (CBD), Cannabidiolic Acid (CBDA), Cannabigerol (CBG), Cannabigerolic Acid (CBGA), Cannabinol (CBN) were from Sigma Aldrich.

### Calcium assay (bulk method)

Cells were washed and incubated with 0.2 micromolar Fluo-4 [54] for 30 min at 37°C in a standard modified Ringer’s solution of the following composition (in mM): NaCl 145, KCl 2.8, CsCl 10, CaCl_2_ 10, MgCl_2_ 2, glucose 10, Hepes·NaOH 10, pH 7.4, 330 mOsm. Cells were transferred to 96-well plates at 50,000 cells/well and stimulated as indicated. Calcium signals were acquired using a Flexstation 3 (Molecular Devices, Sunnydale, USA). Data were analyzed using SoftMax® Pro 5 (Molecular Devices). Where indicated, nominally calcium-free external conditions were achieved by the preparation of 0mM CaCl_2_ Ringer solution containing 1mM EGTA.

### Electrophysiology

Patch-clamp experiments were performed in the whole-cell configuration at 21–25°C. Patch pipettes had resistances of 2–3 MΩ. Data were acquired with PatchMaster software (HEKA, Lambrecht, Germany), controlling an EPC-10 amplifier. Voltage ramps of 50 ms spanning the voltage range from −100 to 100 mV were delivered from a holding potential of 0 mV at a rate of 0.5 Hz, typically over a period of 180 s (3 min). Voltages were corrected for a liquid junction potential of 10 mV. Currents were filtered at 2.9 kHz and digitized at 100 μs intervals. Capacitive currents were determined and corrected before each voltage ramp. The current development graphs were generated by extracting currents at the voltages of −80 mV and +80 mV. Data were analyzed with FitMaster (HEKA, Lambrecht, Germany), and IgorPro (WaveMetrics, Lake Oswego, OR, USA). Where applicable, statistical errors of averaged data are given as means ± s.e.m. with n determinations. The activated current amplitudes are analyzed in nA rather than pA/pF. This decision was made due to the high current amplitudes (1 to 6 nA) resulting from the TRPV1 transfection of the channel. Also, the cell size selected for patching was in the 9–12 pF range. We compared the analysis in nA and in pA/pF and found no significant differences mainly due to consistent cell size selection and high current amplitudes in the nA range.

For patch-clamp recordings, HEK293 cells were kept in a standard sodium-based external Ringer’s solution containing (mM): 140 NaCl, 1 CaCl2, 2 MgCl2, 2.8 KCl, 11 glucose, 10 HEPES-NaOH with a pH of 7.2 and osmolarity of 300 mOsmol. To assess the effects of external Calcium (Ca) on TRPV1 inactivation kinetics, Ca at different levels were tested including 0, 1, and 3 mM. In Experiments with zero external Ca, EGTA 10 mM was added and the Na concentration was lowered to 130 mM to maintain standard osmotic conditions at 300 mOsmol. For rapid external solution application and exchanges, we used the SmartSquirt delivery system (Auto-Mate Scientific, San Francisco CA, USA) that included four cryo tubes allowing for solution exchanges within one patch. This system included a ValveLink TTL interface between the electronic valves and the EPC10 amplifier (HEKA, Lambrecht, Germany). This electronic configuration allowed for programmable solution changes via the PatchMaster software (HEKA, Lambrecht, Germany).

The cytosol was perfused with an intracellular patch pipette solution containing (mM): 140 Cs-glutamate, 8 NaCl, 1 MgCl2, 3 MgATP, 10 HEPES-CsOH. The pH of the pipette solution was adjusted to pH 7.2 and osmolarity measured at 300 mOsmol. The level of free unbuffered Ca in the cytosol was adjusted using the calculator provided with WebMaxC (http://www.stanford.edu/~cpatton/webmaxcS.htm). Cytosol [Ca^2+^]_i_ was buffered to 180 and 620 nM with 10 mM Cs-BAPTA and Ca 4.5 or 7.4 mM respectively, calculated with WebMaxC and as indicated in the text. Whenever 10 mM Cs-BAPTA was added, we lowered the external Cs-glutamate from 140 to 120 mM to maintain consistent osmolarities at 300 mOsmol. When experimental aims required using unbuffered Ca that excluded both BAPTA and Ca (identified in the results as Fca), this absence of buffering allowed for free accumulation of internal Ca that was determined primarily by the permeation of external Ca into the cytosol.

### Analysis

Results are generally shown as the mean ± standard deviation. The electrophysiology results error bars display standard error of mean (SEM). Statistical significance was determined based on Student’s t-test or ANOVA. Adjacent to data points in the respective graphs, significant differences were recorded as follows: single asterisk, p < 0.05; double asterisk, p < 0.01; triple asterisk, p < 0.001; no symbol, p > 0.05. Experiments are all *n* of at least 3.

## Results

### TRPV1 expression confers cannabinoid-dependent calcium fluxes upon HEK293 cells

**Figure 1.**
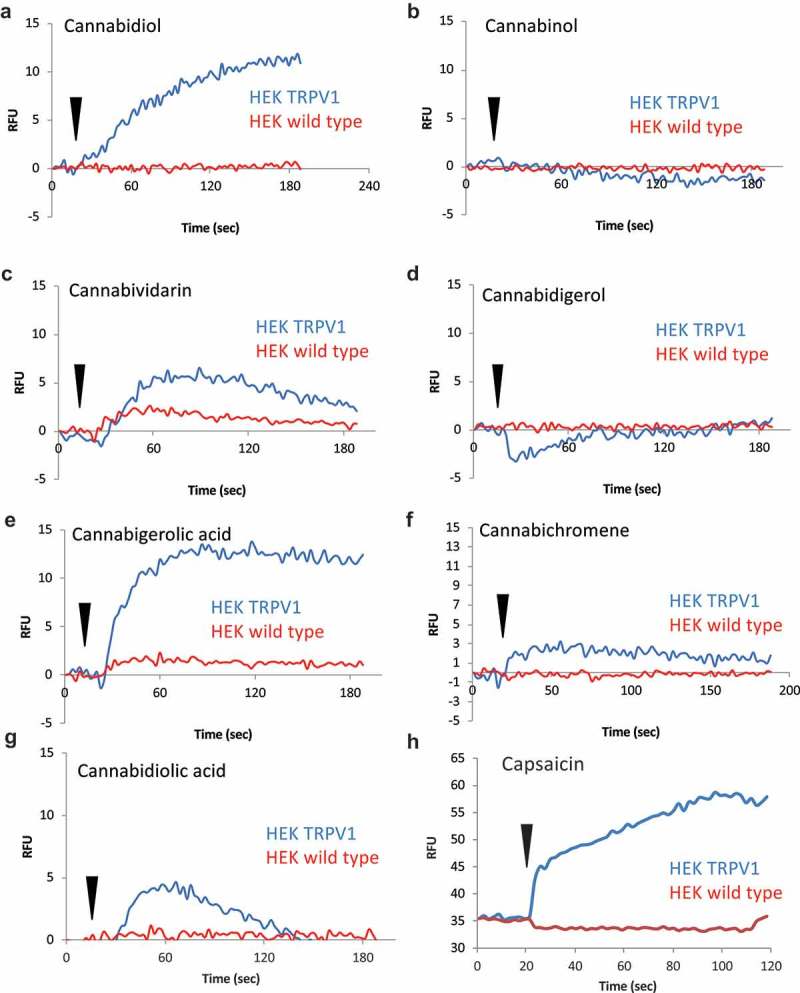
TRPV1-dependent calcium responses initiated by major and minor cannabinoids. A-G. HEK wild type and HEK-TRPV1 expressing cells were loaded with Fluo-4 and population-based calcium assays were performed in a buffer containing 1mM external CaCl_2_. These are population-based (bulk Ca^2+^) measurements with each trace representing averaged triplicates of 100,000 cells per sample. After establishing a baseline for 20 sec in the presence of a matched vehicle, cells were stimulated with the indicated cannabinoid at 10 μM. **H**. Protocol as for A-G but with the TRPV1 ligand, Capsaicin (50 nM) as the stimulus. Capsaicin data were captured during a different experiment run and baselines vary due to dye loading differences between experiments.

We performed a systematic analysis of cannabinoid-induced calcium responses and assessed the role of TRPV1 in these responses. Cannabinoids other than the highly psychoactive tetrahydrocannabinol (THC) that rank order in abundance directly below THC in *Cannabis* chemotypes were selected for analysis. Cannabidiol (CBD), Cannabinol (CBN) and the minor cannabinoids Cannabidiolic Acid (CBDA), Cannabidivarin (CBDV), Cannabichromene (CBC), Cannabigerol (CBG), Cannabigerolic Acid (CBGA) were selected [55,56]. These compounds are variously the subject of biopharmaceutical development, are contained in currently available nutraceutical formulations. In addition, as components of “medical” *Cannabis sativa*, they are currently in use by numerous patients worldwide for disorders such as pain, anxiety, neurodegenerative disorders, and glaucoma. Figure 1A-H shows that each of the tested cannabinoids initiates a calcium flux in HEK-TRPV1 overexpressing cells, with the exception of Cannabigerol and Cannabinol. Comparative responses to a single dose (10 μM) are shown, and these measurements were made in the presence of 1mM external calcium. These are population-based (bulk Ca^2+^) measurements with each trace representing averaged triplicates of 100,000 cells per sample. We noted that in most cases these responses were dependent on the overexpression of TRPV1, with WT HEK293 responding slightly to Cannabividarin and Cannabigerolic acid. For comparison purposes, and confirming the fidelity of the HEK293/HEK-TRPV1 comparison, Figure 1H shows the response to Capsaicin. For each of the compounds, we performed dose responses at the population level. For CBG concentrations of 30–50 μM initiated small calcium fluxes. For CBN concentrations of 30–50 μM initiated small and transient calcium transient fluxes. The lower limit of detectable calcium responses in this system varied from 10-100nM (CBDV, CBGA) to 1–10 μM (others). In separate single cell Ca^2+^ assays using confocal imaging (data not shown) we calibrated responses and were able to estimate absolute changes in Ca^2+^ I for key compounds. Example values of peak Ca^2+^ i for compounds tested 60 s after addition of stimulus at 10 μM are as follows: baseline (vehicle stimulation), 111nM; Capsaicin, 900nM; CBD, 320nM; CBN, 108nM; CBDV, 380nM.

### TRPV1 conductances that are evoked via capsaicin or a major cannabinoid are sensitive to capsazepine

**Figure 2.**
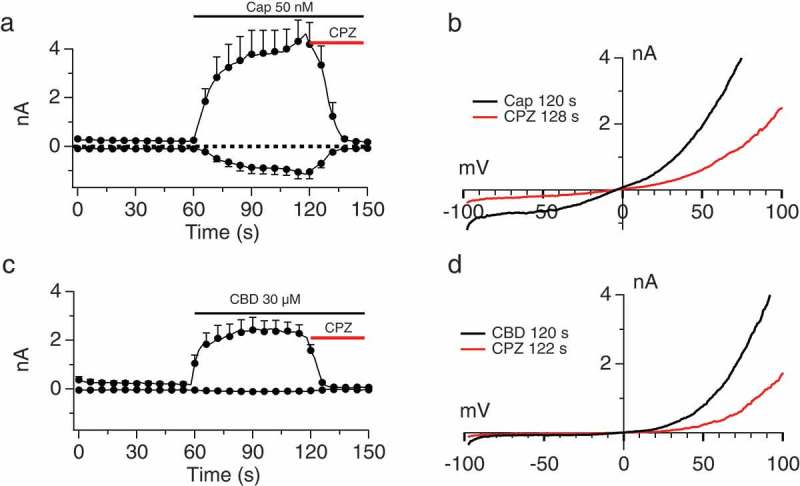
Sensitivity of Capsaicin- and Cannabinoid-induced conductances to Capsazepine. A, B. Current development graph (A) and extracted current/voltage relationship (B) in HEK-TRPV1 stimulated with 50 nM Capsaicin and then application of 10 µM Capsazepine (CPZ). C, D. Protocol as in (A), but with stimulus of 30 μM Cannabidiol (CBD). The recording solutions were Ca 0 internally and externally and the n determinations were from 5 to 6 patches. The current development graphs (A and C) were generated by extracting currents at the voltages of −80 mV and +80 mV.

Bulk calcium measurements such as those in Figure 1 provide a picture of overall responses across a cell population but do not describe specific conductances or response kinetics. We therefore sought to examine the effect of each cannabinoid upon the TRPV1 conductance using whole-cell patch-clamping. We first verified the fidelity of our HEK-TRPV1 expression system for the detection of the TRPV1 conductances. When 50 nM Capsaicin (Cap) was applied for 60 s an outwardly rectifying TRPV1 current was recorded while application of 10 µM Capsazepine (CPZ) [57] reduced both the inward and outward current during a subsequent 30-s application (Figure 2A and B). We then applied 30 µM CBD to the same TRPV1 overexpression system and recorded an outwardly rectifying current that was reduced with CPZ (Figure 2C and D). These data suggest that this expression system is reporting TRPV1 currents which are responsive to both Capsaicin and cannabinoids such as CBD.

### Diverse TRPV1 activation responses initiated by CBD, CBN, CBDV, and CBG

**Figure 3.**
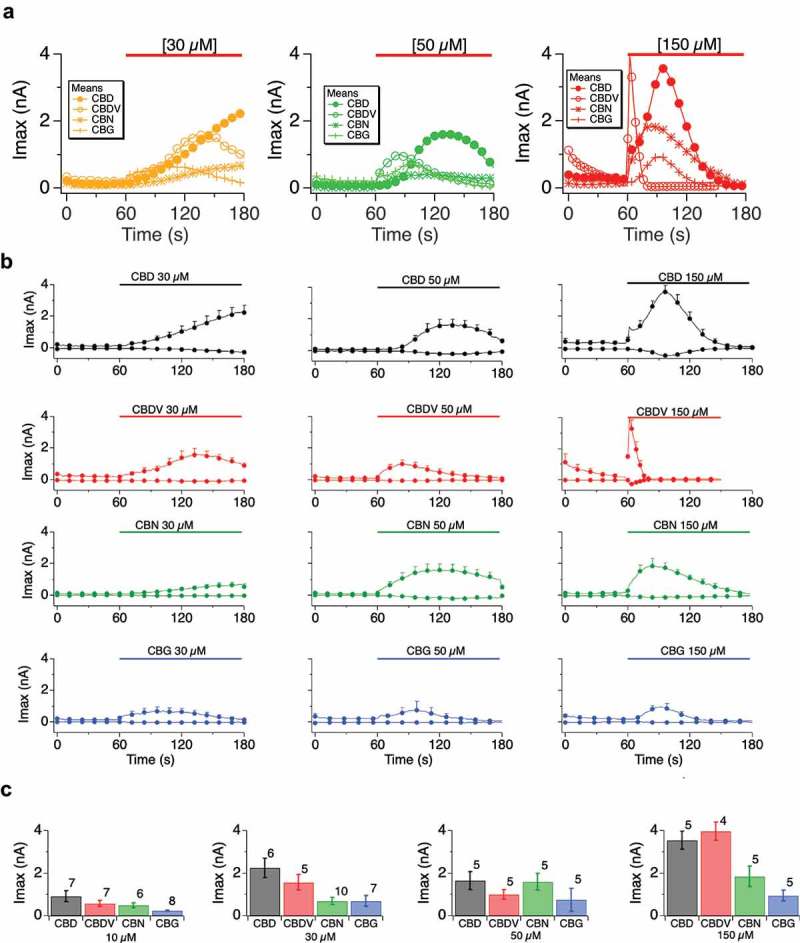
Comparative TRPV1 responses to different cannabinoids. A. Current development over time (Imax, outward) for HEK-TRPV1 exposed for 120 s, starting at 60 s and ending at 180 s, to the indicated cannabinoids of CBD (closed circle), CBDV (open circle), CBN (asterisk) and CBG (cross). The color code for cannabinoid dose were: at 150 µM (red), 50 µM (green) and 30 µM (yellow). Panel **B**. data as in (**A**) by compound and by dose, individual; responses showing inward currents (from −80 mV) and outward currents (from +80 mV). **C**. Histogram summary of Imax data from (**B**). Recording conditions were NaR, Ca 1 mM with unbuffered internal calcium (Fca) and the n determinations varied from 5 to 8 patches and as indicated in panel C, bar graphs.

At this point in the study, we focused upon four cannabinoids, which exemplified the range of behaviors we observed at TRPV1. Figure 3A presents compiled dose responses at 30, 50 and 150 μM applications of CBD, CBN, CBDV, and CBG. These responses were measured in unbuffered internal and external calcium. This absence of buffering allowed for free accumulation of internal Ca that was determined primarily by the permeation of external Ca into the cytosol. We observed that the kinetics of the activation of TRPV1 via cannabinoid application is affected by dose and by cannabinoid type (broken out in Figure 3B showing the individual Imax over time for each dose and each cannabinoid, displaying the distinct activation and inactivation kinetics that we observed). In Figure 3B, as the dose is increased the activation speed is accelerated as well as speed of the deactivation. As the dose increases, the activation kinetics or the time to reach maximal current peak is accelerated and the less amount of time is spent at peak current. Figure 3C shows histograms of the attained Imax for each compound by dose. The maximal attained current is also variant between the different cannabinoids (Figure 3C) with typical Imax ranging from 1 to 4 nA depending upon the dose and the compound.

### EC50 for cannabinoid activation of TRPV1

**Figure 4.**
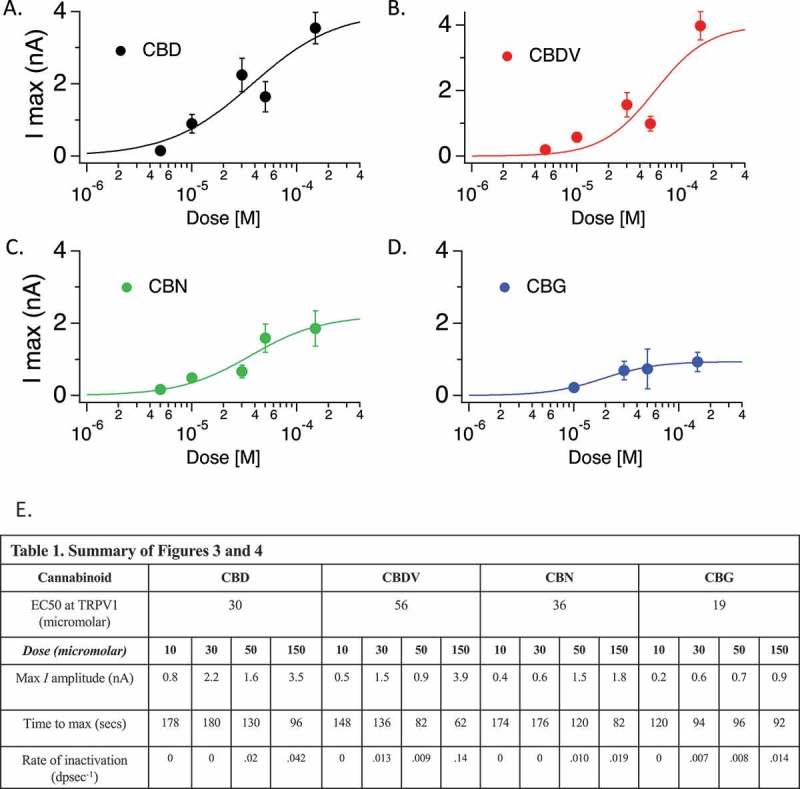
Cannabinoid dose response and EC50 calculation for TRPV1. A-D. Hill plots for the indicated cannabinoids across dose rages tested. **E. Table 1**. Summary table of data from Figures 4 and 5. This table summarizes calculated EC50, and several features of the conductances at TRPV1 by compound and by dose.

We assessed the Imax and mean Imax per dose for the four cannabinoids, and used these data to calculate EC50. Figure 4A-D show Hill diagrams for each of these compounds and Figure 4E presents the calculated EC50 under our experimental conditions. We note that these are likely to be over-estimates due to the lability of cannabinoids in both storage and during application and most likely that under certain conditions (cannabinoid species and dose) the inactivation kinetics may underestimate the current amplitude steady-state level.

**Figure 5.**
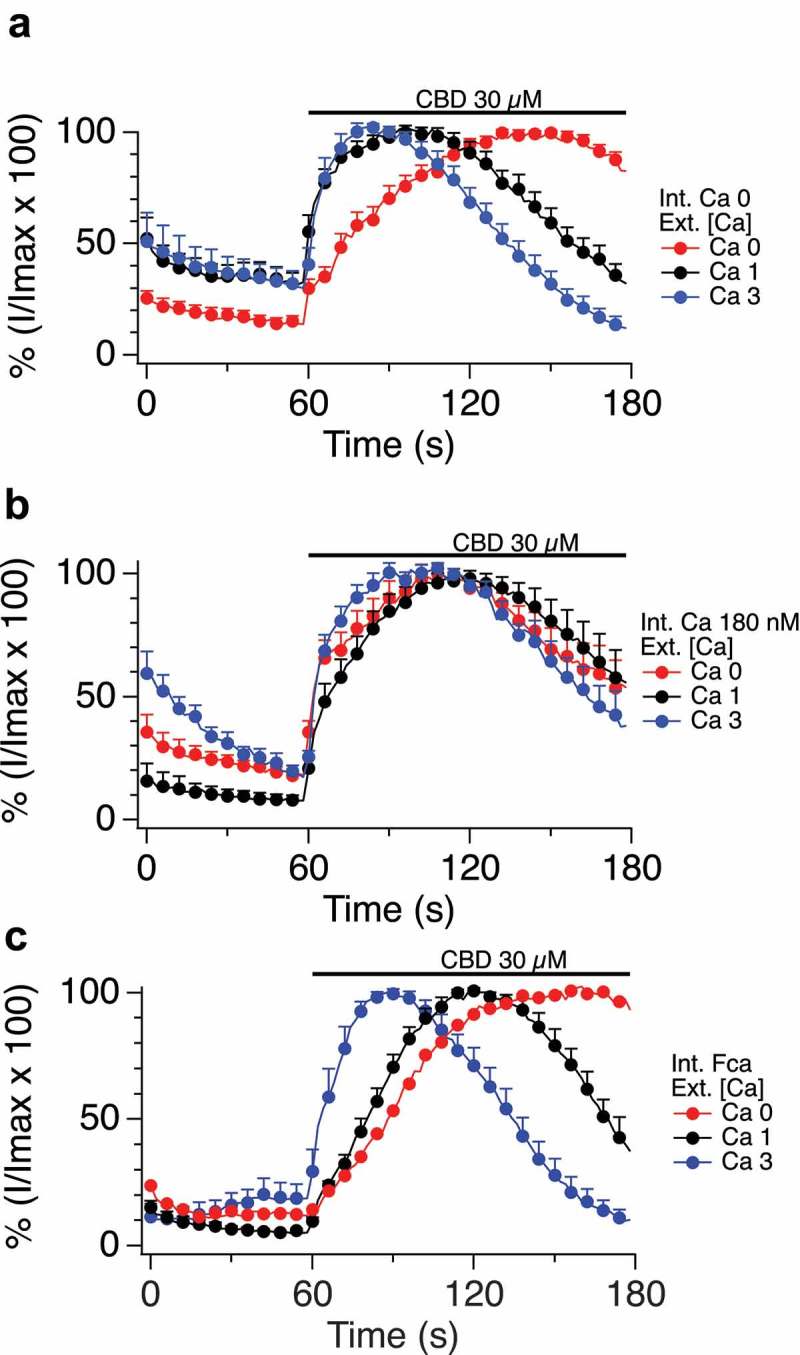
A-C. Impact of altered internal and external calcium levels upon CBD-induced TRPV1 currents. Normalized current development graphs (percentage of Imax) for CBD at the indicated doses, recordings performed on 0, 1 or 3 mM external calcium with internal calcium buffered to zero, 180nM or allowed to be determined by influx (FCa, free calcium). **D, E**. Comparison of CBD and CBN responses in constant external calcium with varying internal calcium levels from Ca 0 to Ca 180 nM, 620 nM and FCa. **F-I**. Comparison of CBD and CBDV at low and high doses in 0 and 1mM external calcium with internal calcium buffered to 0, 180 nM, 620 nM, and FCa. The n determinations for Figure 5 (panels A to I) varied from 6 to 14 patches. **J. Table 2**. Figure 5 data summary.

**Figure 5.**
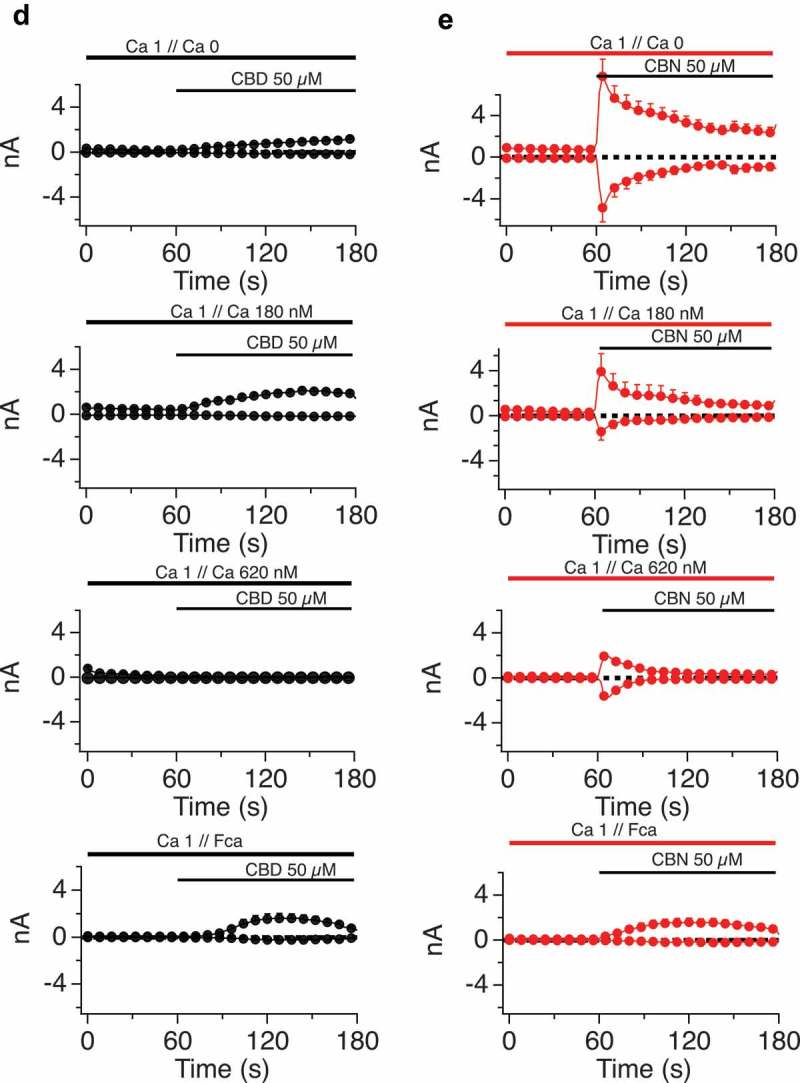
(Continued).

**Figure 5.**
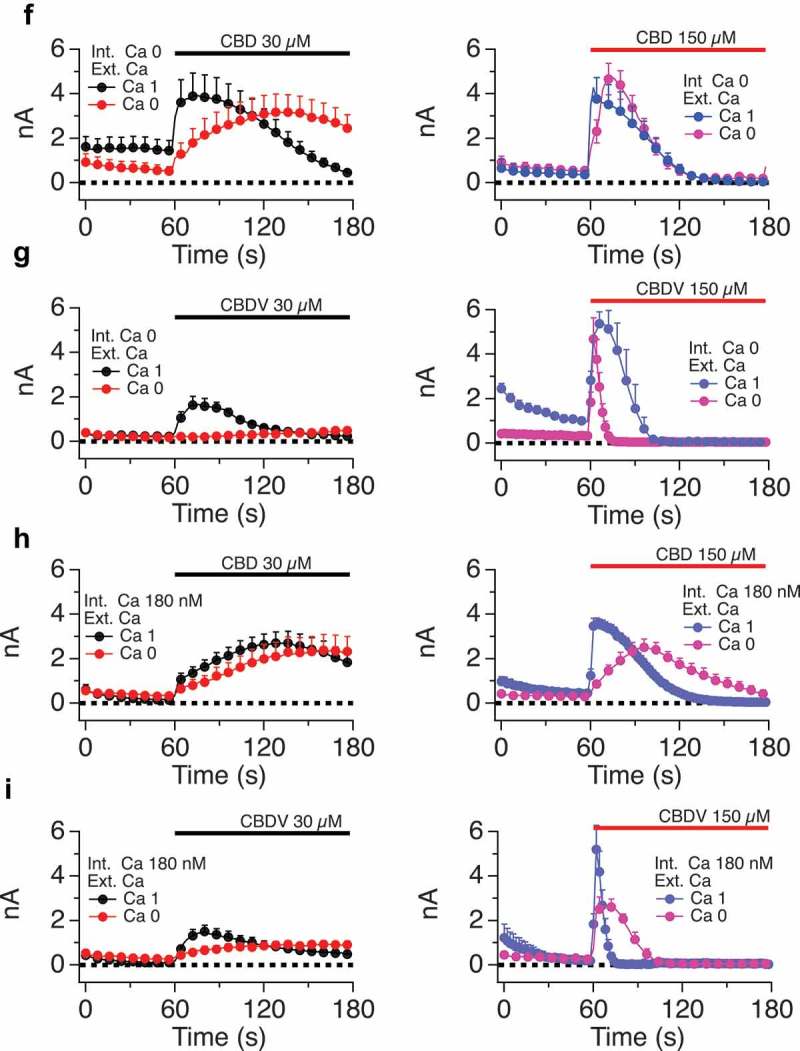
(Continued).

**Figure 5.**
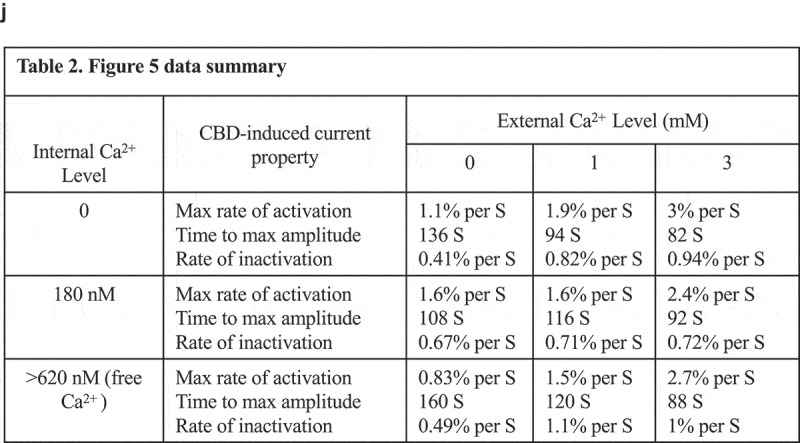
(Continued).

### Cannabinoid regulation of TRPV1 exhibits dependence on external and internal calcium concentrations

At this point in the study, we focused primarily on cannabidiol (CBD), in order to explore the regulation of the physiological properties of TRPV1 by a cannabinoid. We first examined the dependency of CBD responses on external calcium levels, while buffering internal calcium to a constant 180 nM (which is close to resting cytosolic levels), buffering internal calcium to zero, or leaving internal calcium levels unbuffered (Fca). Figure 5A-C presents time courses of current Imax with external calcium at 0, 1 or 3mM under each of the three internal buffering conditions. With any constant internal condition, external calcium influences the activation time (lag) and inactivation kinetics of the TRPV1 responses. Figure 5A shows that lower external calcium levels slow activation kinetics (due to the current carrying contribution of the calcium ions) and lead to far slower inactivation once maximal currents are obtained. By contrast, increasing external Ca levels from 1 and 3 mM shows no effect on activation kinetics but results in the acceleration of the inactivation kinetics. Figure 5B (constant internal Ca 180 nM) presents these data normalized to Imax, with external calcium constant for each panel but varying internal calcium. These data also highlight the differences in current development when sodium (0 Ca_ext_, 0 Ca_int_) rather than a mix of sodium/calcium ions are flowing through the non-selective cation channel TRPV1.

Figure 5D, with no calcium buffering, demonstrates that increasing external Ca levels accelerates the activation kinetics followed by more rapid inactivation kinetics. Figure 5D E explores the effect of internal calcium levels on shaping the kinetics of responses to two cannabinoids, CBD (Figure 5D) and CBN (Figure 5E). Under constant external calcium conditions and constant doses of the respective cannabinoid, we observe that higher internal calcium levels (from Ca 0 to Ca 180 nM, 620 nM, and unbuffered Fca) are associated with lower attained maximal currents and faster inactivation of currents that do develop. The documented cytosolic calcium-dependent inactivation of TRPV1 appears to be at play. CBD (Figure 5D) causes gradual current development, which inactivates only when internal calcium is buffered above zero. For example, when internal Ca is buffered to 620 nM the TRPV1 channel becomes totally inactivated. CBN (Figure 5E) causes a different presentation of TRPV1, which activates quickly but rapidly inactivates with slower kinetics, presumably in relationship to the amount on calcium entering via the channel. In some cases (CBD and CBN at 620nM internal or unbuffered internal calcium), this inactivation seems to outpace current development, leading to inactivation concurrently canceling out the channel’s flux. Figure 5F-I compares the effect on internal calcium levels on responses to CBD and CBDV at low and high doses. Again, high doses inactivate faster associated with increased availability of calcium in the cytosol. Table 2 summarizes the data from Figures 4 and 5.

### Cannabinoid regulation of TRPV1 is differentiated from capsaicin responses by a lack of attainment of a pore-dilated state

**Figure 6.**
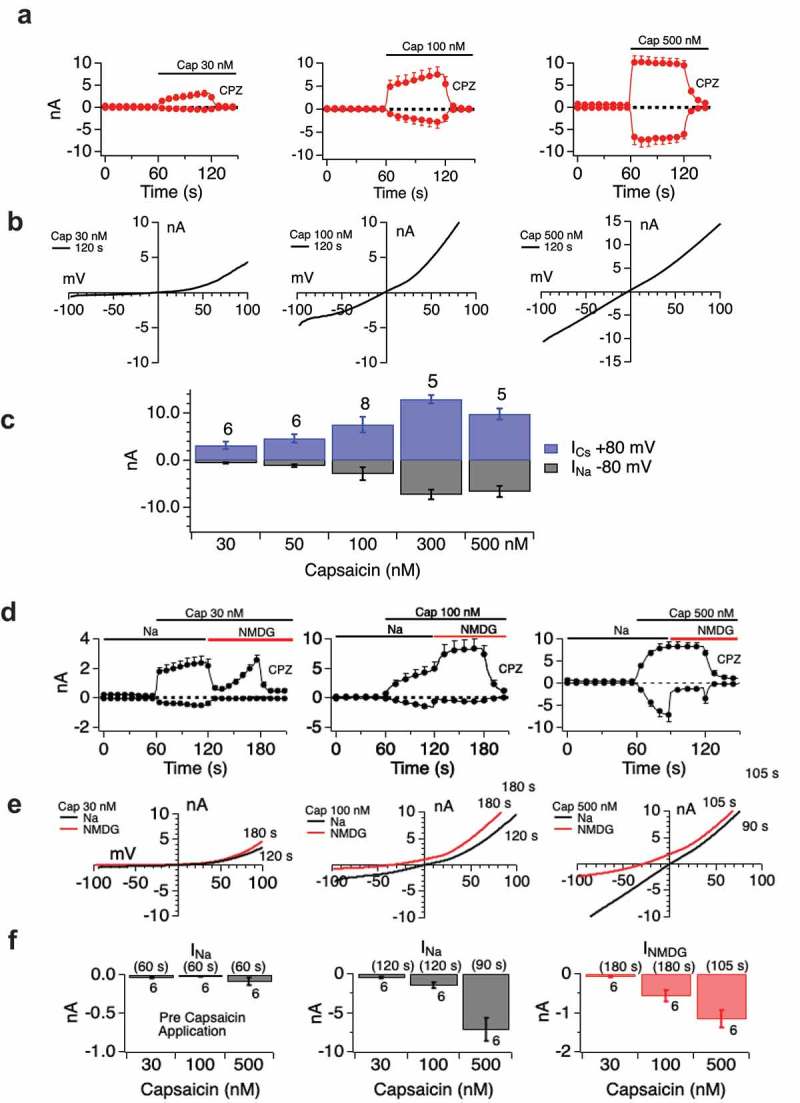
Two state nature of TRPV1. Panels A-D. Dose-dependent attainment of pore-dilated state by TRPV1 in response to Capsaicin. **A**. Current development over time with the indicated Capsaicin doses. **B**. Extracted I/V curves showing transition from rectifying to non-rectifying state with increasing dose/current amplitude. **C**. Histogram summary of inward and outward current portions per dose. Histogram data confirm that linearized I/V relationship corresponds to pore-dilated state by demonstrating increased Na current amplitude. Current development relationships (**D**) suggest some pore-dilation (NMDG sensitivity) even at the lowest Capsaicin doses. Current/voltage relationships (**E**) and I max histograms for sodium and NMDG permeation (**F**) illuminate the distinct states.

TRPV1 is a two-state channel. With Capsaicin activation it passes through a rectifying state rapidly, then attains a non-rectifying, pore-dilated state characterized by a linear I/V relationship and high level of permeability, including small cations such as Na to large cations such as NMDG [58–61]. This pore-dilation leads to sustained and high permeation characteristics of the channel to Capsaicin, which are important drivers of neuronal activation and eventual desensitization of the neuron due to unfettered calcium and sodium entry. We examined the two-state behavior of TRPV1 in response to cannabidiol. First, with Capsaicin we established the two-statenature of the channel in our experimental system. Figure 6A-C shows that low and medium doses of Capsaicin result in rectifying currents but that at a higher Capsaicin dose a non-rectifying current is observed. Both states (non dilated and dilated state) of the current are sensitive to Capsazepine. Figure 6C summarizes the inward and outward current sizes at a range of Capsaicin doses (30, 10 and 500 nM). Figure 6D-F documents the increasing permeation of NMDG as the rectifying nature of the channel decreases, and TRPV1 attains its dilated state, demonstrating that the linearized I/V relationship is indeed a marker of the dilated (NMDG-permeant) state.

We explored state transition in response to a representative cannabinoid, CBD. Figure 7A-D shows that even at high attained Imax, and at high induction times, the TRPV1 currents remain rectifying and sensitive to CPZ. Even when currents attain ~10 nA there is no transition to the pore-dilated state in response to CBD. Indeed, across induction times of 0–25 h and CBD doses of up to 150 μM, we saw only one recording where CBD caused a linear non-rectifying current to develop and that was in a cell with a large break-in current (data not shown). Figure 7E, F and 7G, H shows a similar lack of attainment of the pore-dilated state for CBG and CBDV in addition to CBD.

**Figure 7.**
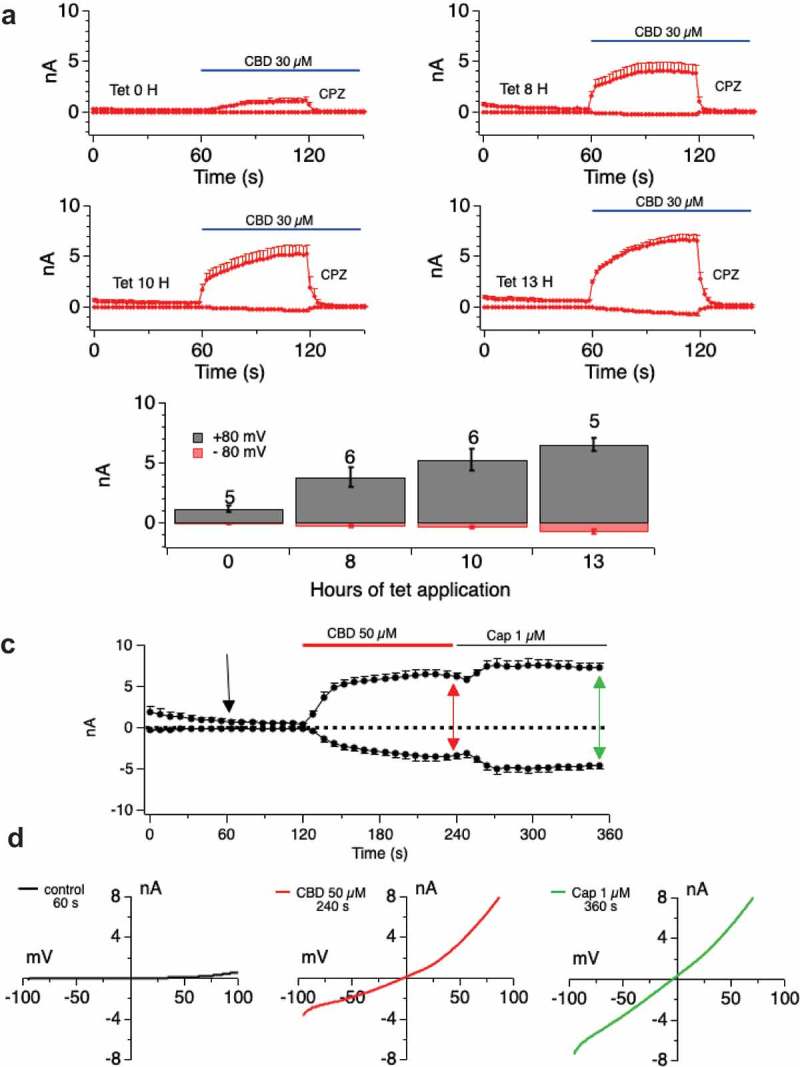
Examination of pore-dilation in Cannabinoid-evoked TRPV1 currents. **A, B**. Driving large CBD-evoked currents through extended tetracycline induction time (current development graphs shown in A for tetracycline inductions of 0–13 h) does not translate to attainment of a non-rectifying pore-dilated state for the conductance (B). **C**. Currents evoked by CBD and Capsaicin of similar amplitudes are distinguished by their current/voltage relationships. Induction time of 25 h with CBD doses of 50 μM followed by 1 μM Capsaicin. I/V relationships (**D**) show that CBD-evoked currents are rectified while Capsaicin-evoked current is highly linear. **E, F, G. H**. Protocol as in Figure 7 **A-D** but with CBDV (**E, F**) or CBG (**G, H**).

**Figure 7.**
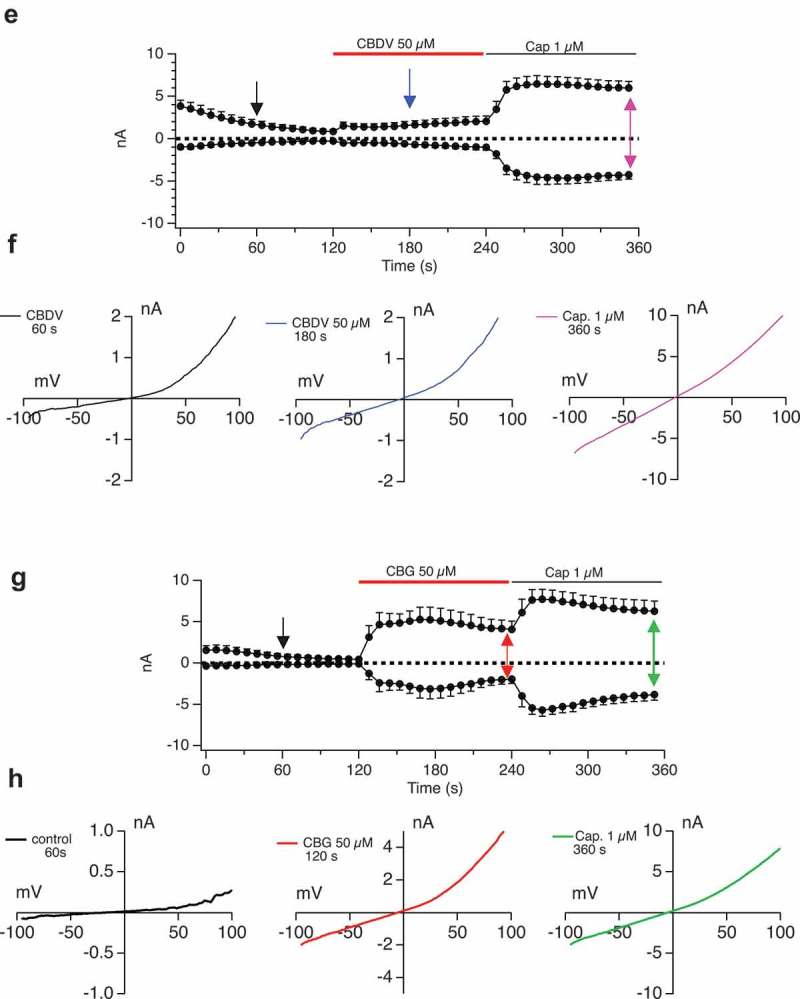
(Continued).

### Differential to responses cannabinoids by TRPV2, TRPM8, and TRPA1

The potential for different cannabinoids to target specific TRP channels or to co-target more than one channel type, is both of potential therapeutic interact for pain [35]. We used overexpression systems for TRPV2, TRPM8, and TRPA1 to compare the impact of each cannabinoid in a side-by-side fashion. Figure 8 A-U and Table 3 summarize these responses using bulk calcium assays. These are population-based (bulk Ca^2+^) measurements with each trace representing averaged triplicates of 100,000 cells per sample. There are clear differences in responsiveness between cannabinoids at a single channel type and between channel types to a given cannabinoid. Once these data and the data presented above on TRPV1 are extended electrophysiologically, they can provide a foundation for rational design of therapeutic strategies on the basis of response kinetics, desensitization, and receptor selectivity.

**Figure 8.**
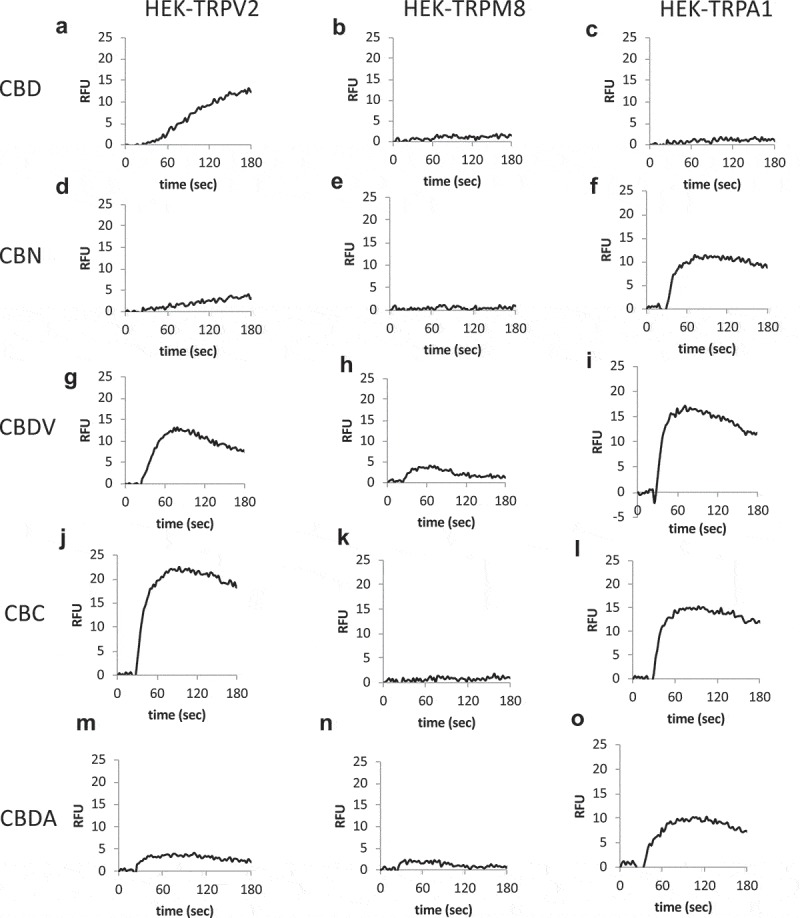
Cannabinoid responses in cells overexpressing nocioceptive TRPs. A-U. HEK-TRPV2, TRPA1, or TRPM8 expressing cells were loaded with Fluo-4 and population-based calcium assays were performed in a buffer containing 1mM external CaCl_2_. After establishing a baseline for 20 sec in the presence of a matched vehicle, cells were stimulated with the indicated cannabinoid at 10 μM. These are population-based (bulk Ca^2+^) measurements with each trace representing averaged triplicates of 100,000 cells per sample. V. Table 3. Summary data for Figure 8.

**Figure 8.**
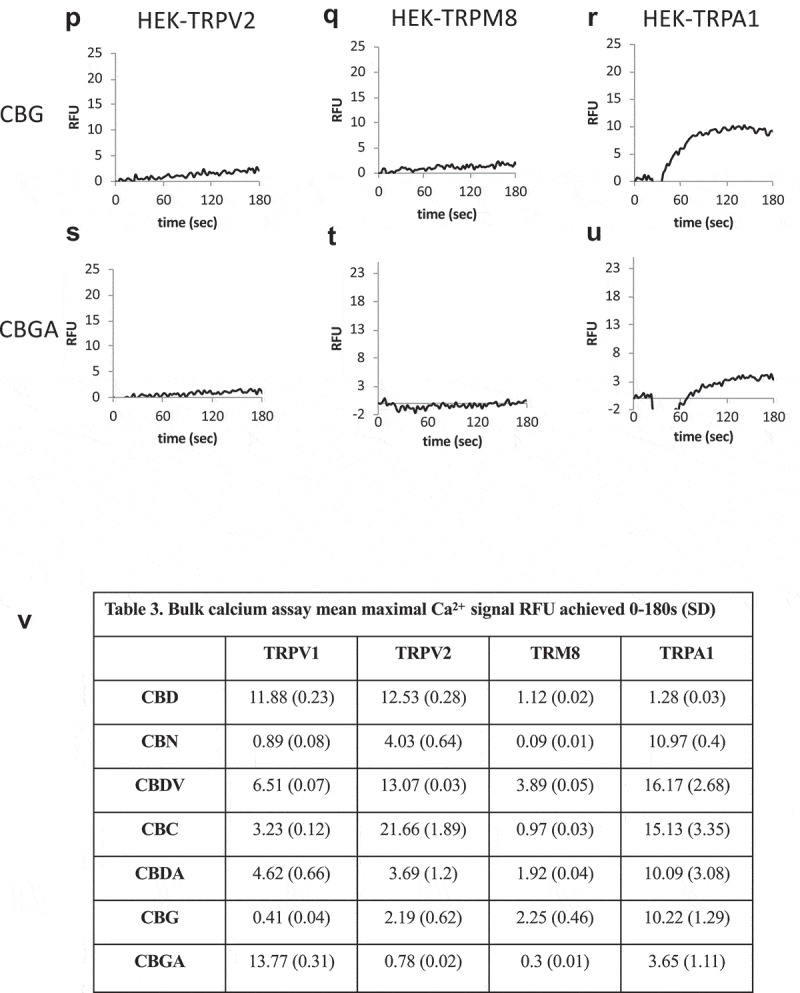
(Continued).

## Discussion

This study presents an analysis of TRPV1 physiology in response to a suite of cannabinoid compounds. We noted differential activation and inactivation kinetics for the various compounds, and that the various compounds exhibited quite different Imax. Dependency of attained current upon internal and external calcium levels was observed, as has been previously reported for Capsaicin [62–65]. However, given the very different kinetics and current development profiles of the cannabinoid-evoked currents, there is a concomitant difference in the shaping of the currents by internal calcium-mediated inactivation between cannabinoids and Capsaicin. One major area of contrast between the cannabinoid and Capsaicin-evoked currents is in the lack of apparent pore-dilation in response to the former, even when large currents are driven by high doses of cannabinoids or long induction times for the channel protein. Taken together, these various findings suggest that cannabinoids are viable potential analgesics and that one or more of these compounds may contribute to the patient-reported analgesic effects of *Cannabis sativa* that is used in a pseudo-medical setting.

Capsaicin cream is a therapeutic standard for topical TRPV1 activation and pain desensitization. However, therapeutic application of Capsaicin as a topical pain treatment results in high levels of initial discomfort prior to desensitization [45,49,51,66–68]. We and others show that Capsaicin activates the TRPV1 channel into the dilated state within a few seconds [59]. In contrast, TRPV1 exposure to the cannabinoids activates the channel primarily into state 1 (the non-dilated state). If there is a connection between the pore dilation and large amounts of sodium entry that results in neuronal death and tissue desensitization, then this finding may have implications for topical pain therapy. Specifically, it would need to be explored whether cannabinoid-based analgesics would outperform or under-perform Capsaicin in topical formulations. A cannabinoid with sustained, non-dilated, TRPV1 channel activity would potentially be an effective analgesic but if state 2 is required for neuronal cell death and tissue desensitization then it may not achieve that goal. However, this type of cannabinoid may be a highly effect desensitizer at the cellular level, initiating TRPV1 internalization as effectively as Capsaicin. Clearly, some of the next experimental steps need to be a comprehensive analysis of desensitization at both the cellular (responses to repeated sequential doses of the ligand) and tissue (induction of neuronal cell death in sensory bundles) levels [69]. Since agonist-mediated TRPV1 desensitization as an approach has been beset by issues of burning sensation side effects in response to topical Capsaicin, it is possible that the within the types of TRPV1 response initiated by cannabinoids there is an approach (dose, type of cannabinoid) that separates excitatory and analgesic effects. Moreover, burning sensations are not typically associated with cannabis exposure, even with relatively high percentage CBD topical formulations in widespread nutraceutical use.

Cannabinoids bind to two types of receptor at the cell membrane, the metabotropic GPCR (CB1, CB2, GPR55) and the ionotropic TRP channels (V1, V2, M8, A1) [37,41]. Our assumption in the experiments shown here is that the response we see represents direct action of the cannabinoid at TRPV1, rather than a coupled GPCR-ion channel response seen in other metabotropic/ionotropic pairs such as the glutamate receptors [70]. We find that Capsazepine (a competitive antagonist of Capsaicin [71]) also competes cannabinoid activation, suggesting similar competition. In addition, we explored the potential for additive effects of Capsaicin when added sequentially after each cannabinoid (not shown). Cannabidiol activates large V1 currents, to which Capsaicin is unable to behave additively (most or all receptors are therefore continuously engaged by CBD). These CBD currents are sustained (not rapidly inactivating). Cannabinol activates large V1 currents. These CBN currents are rapidly inactivating (our other data show this is a calcium-dependent inactivation and so subsequent Capsaicin activation behaves additively). CBDV activates large sustained V1 currents to which Capsaicin behaves additively. CBG activates large sustained V1 currents, to which Capsaicin behaves only slightly additively. Again, these data speak to common targeting of TRPV1 between Capsaicin and cannabinoids but with different physiological outcomes.

Cannabinoids are of significant interest in the context of “medicinal” *Cannabis* use. Pain is one of the most common indications for which medical marijuana is legally allowed to be prescribed and is demanded by patients. The psychoactive nature of THC-containing whole chemovars of *Cannabi*s, which is typically the available form of the drug in dispensaries, leads to regulatory issues and adverse side-effects. Moreover, issues of contamination (pesticides, metals, microbial), inconsistency and chemovar misidentification beset patients who present at dispensaries [56]. The rigorous evaluation of individual cannabinoid physiology at a defined target, such as TRPV1, is a concrete step towards the rational design of single cannabinoid or cannabinoid sub-mixtures formulations that have low side effect profiles, can be produced in a regulated manner, and which are efficacious. The distinct response profiles of the different cannabinoids that we observe also provide the possibility of fine-tuning or shaping desirable responses using cannabinoid mixtures. At the level of the sensory neuron bundles, the fact that cannabinoids appear to discriminate between TRP receptors and that the receptors in turn respond distinctively to the compounds, again offers the potential for rational design of therapeutic mixtures.
